# Successful use of tapering strips for hyperbolic reduction of antidepressant dose: a cohort study

**DOI:** 10.1177/20451253211039327

**Published:** 2021-08-27

**Authors:** Peter C. Groot, Jim van Os

**Affiliations:** User Research Centre Netherlands, UMC Utrecht Brain Centre, University Medical Centre Utrecht, Utrecht, The Netherlands; Department of Psychiatry, UMC Utrecht Brain Centre, University Medical Centre Utrecht, Postbus 85500, 3508 GA Utrecht, The Netherlands; Department of Psychiatry and Neuropsychology, Maastricht University Medical Centre, Maastricht, The Netherlands; Department of Psychosis Studies, Institute of Psychiatry, King’s College London, King’s Health Partners, Institute of Psychiatry, London, UK

**Keywords:** antidepressants, dependence, drug withdrawal symptoms, patient medication knowledge, tapering

## Abstract

**Background:**

Tapering strips facilitate antidepressant discontinuation, allowing for personalised titration of discontinuation to the intensity of withdrawal. A tapering strip consists of antidepressant or other medication, packaged in a 28-day roll of daily pouches, each with the same or slightly lower dose than the one before. Previous studies demonstrated 70% real-world effectiveness of tapering strips. Here, we present a third, questionnaire-based retrospective cohort study in a large sample.

**Methods:**

Patients whose doctor had prescribed tapering strips between October 2015 and December 2018 were sent a questionnaire for participation after completion of tapering between December 2015 and January 2020. Of 1240 individuals who returned a questionnaire (response rate: 59%), 987 (80%) used an antidepressant, of whom 824 (83%) had wished to discontinue their antidepressant.

**Results:**

The sample was demographically representative of antidepressant users in the Netherlands. Less than 40% of participants had heard of tapering strips through their clinicians – Internet was the most frequent source. Of the 824 individuals, 341 (41%) had used strips for tapering venlafaxine, 206 (25%) for paroxetine and 277 (34%) for other antidepressants. Median duration of antidepressant use was 5–10 years, and most (71%) had tried to come off without tapering strips at least once. Most patients (72%) were able to discontinue their antidepressant, using a median of two strips to taper over a median period of 56 days. Females and individuals with (1) more severe experience of withdrawal during the use of tapering strips, (2) more years of use of antidepressant medication and (3) more previous attempts at discontinuation were less likely to be able to discontinue their antidepressant medication with tapering strips.

**Conclusion:**

The results of this study validate, for the third time, the observation that tapering strips can address the problem of antidepressant withdrawal symptoms in individuals attempting to discontinue antidepressants.

## Introduction

Although differences between studies make it difficult to give a precise summary estimate of incidence, there is consensus that users of antidepressants and especially of SSRIs (Selective serotonin reuptake inhibitors) and SNRIs (Serotonin and norepinephrine reuptake inhibitors) often experience difficulties coming off medication.^1,2^ Post-acute withdrawal or protracted withdrawal syndromes from antidepressants can be severe and long-lasting, and its manifestations clinically heterogeneous.^3^ Given high rates of long-term prescribing, particularly in deprived areas,^4^ withdrawal and inability to discontinue antidepressant medication represent a significant public health problem,^5^ particularly given persisting doubts about real-world effectiveness^6^ and indications that continued drug treatment with antidepressant medications may stimulate processes that run counter to the initial acute withdrawal effects of a drug (the oppositional model of tolerance).^7^ Patient surveys suggest current inadequacy of healthcare systems to recognise and manage prescribed drug withdrawal with tapering medication, and patient feedback,^5,8,9^ which is matched by research showing that only a small minority of general practitioners feel sufficiently knowledgeable about withdrawal.^10^ As a result, tens of thousands of patients resort to social media groups that are filling the void left by health services.^5^

A glaring omission in mental health services has been the absence of effort, or even reluctance,^9^ to develop and provide adequate tapering medication.^5,8,9^ There is consensus that, practically, antidepressant withdrawal may be viewed through the lens of drug withdrawal, in general, such as withdrawal associated with benzodiazepines and opiates. This view has much to offer given the solid evidence base supporting treatment of withdrawal by gradual and personal tapering of the substance in question, titrated against the degree of withdrawal discomfort. The recently described Horowitz–Taylor method of withdrawal represents such a common sense strategy.^11^ It remains difficult to implement for antidepressants, however, given that virtually all medications come in dosages that do not allow for flexible and personal tapering.^9^ Although some antidepressants can be cut or come in liquid form, these methods are cumbersome and imprecise^12^ as they were not specifically developed for tapering and have not been investigated for this purpose.

The Horowitz–Taylor method for personalised tapering of psychiatric medication recognises that tapering should be ‘hyperbolic’ to achieve a linear reduction of receptor occupancy to prevent withdrawal,^11,13^ which is otherwise more likely to occur, especially at the end of a taper when lower than registered dosages are required, which were and are still not provided by pharmaceutical companies.^9^ Hyperbolic means that the steps by which the dose is lowered are made smaller and smaller as the dose decreases.^11,13^ Hyperbolic tapering is essentially what many patients, implicitly and without using the word hyperbolic, have been advocating for many years and have tried to achieve themselves by applying do-it-yourself pharmacotherapy.^5,14–16^ Hyperbolic tapering has also been implicitly advocated by some professionals^17,18^ and it was the basic idea behind the development of tapering medication.^16,19^

In recent years, in response to antidepressant users who wished to taper off their medications, the not-for-profit organisation Cinderella Therapeutics in the Netherlands oversaw the development of personal tapering strips for hyperbolic reduction of antidepressant medication in those suffering withdrawal or deemed at risk.^9,19^ A tapering strip consists of antidepressant medication, packaged in a roll or strip of small daily pouches. Each pouch is numbered and has the same or slightly lower dose than the one before it. Strips come in series covering 28 days and patients can use one or more strips to regulate the rate of dose reduction over time in a flexible and personalised manner. Dose and day information printed on each pouch allow patients to precisely record and monitor the progress of their reduction.^9,19^ Also available are stabilisation strips, which can be prescribed to continue the patient on a certain dose for a while, when withdrawal symptoms occur during a taper. This gives the patient time to recover before a more gradual continuation of the taper is initiated.

In two previous studies, we reported that 71% and 66% of patients using a tapering strip, mainly for venlafaxine and paroxetine, were able to come off their drug in both the short term (71%) and the long term (66%), using a median of two 28-day tapering strips.^20,21^ Here, we report an attempt to provide a third replication on the outcome of antidepressant tapering strips in a non-overlapping sample of 824 patients who used tapering strips to discontinue their antidepressant medication. Main outcome was the proportion of these 824 users of tapering strips who indicated they were off antidepressant drugs after completion of the last tapering strip. Conceptually, the study was a retrospective cohort study with assessment of binary outcome (yes/no use of antidepressant) in a cohort with recorded evidence of use of tapering strips in the past, using type of antidepressant as additional exposure of interest. In addition, guided by previous work,^1,2,20^ we tested for association with hypothesised associated factors including duration of antidepressant use, number of previous attempts with tapering strips, level of withdrawal and demographic factors.

## Methods

Whenever a doctor prescribes tapering strips, the patient is invited to provide anonymous feedback for routine quality assessment and feedback after completion of the last prescribed tapering strip. The current sample had thus been invited, between October 2015 and December 2018, to participate after completing their tapering trajectory, between December 2015 and January 2020. A tapering trajectory indicates the amount of time used, and the specific tapering schedule followed, by a participant using one or more tapering strips. Of 1240 Dutch individuals who returned a questionnaire (response rate: 59%), 987 (80%) used an antidepressant, of whom 824 (83%) had wished to discontinue the medication.

Individuals who had attempted tapering of a named antidepressant were included in the analysis (*n* = 824). Under Dutch law, medical ethical approval is not required for analysis of anonymous routine quality assessment data. Patients were given information about the purpose of the questionnaire and were informed that if they wished to consent to participation in anonymised research, they could send back the completed questionnaire. No identifiers were collected and no reminders were sent. There was no overlap between the current sample and the two previously reported samples.^20,21^ The questionnaire was the same as the one that was used in the first published study.^21^

Variables collected were: (1) age (in years), (2) sex, (3) type of medication and duration of use prior to tapering, (4) number of attempts to stop medication before using tapering strips, (5) severity of withdrawal symptoms during previous discontinuation attempts without tapering strips on a 1–7 Likert scale (‘Did you experience withdrawal symptoms?’ 1 = ‘not at all’ and 7 = ‘very much’), (6) severity of withdrawal symptoms experienced with tapering strips on the same 1–7 Likert scale, (7) how well tapering had gone during previous attempts on a 1–7 Likert scale (‘how did the tapering go?’ 1 = ‘very well’ and 7 = ‘very badly’), (8) how well tapering had gone while using tapering strips on the same 1–7 Likert scale and (9) whether or not the individual had tapered off the medication completely. This dichotomous outcome (*n* = 568, 72%; *n* = 31 missing values) will hereafter be referred to as ‘successful tapering’.

### Analyses

Tapering strips were prescribed for a wide variety of mainly antidepressant medications (Table 1). In the analyses, these were reduced to three groups: venlafaxine (*n* = 341, 41%), paroxetine (*n* = 206, 25%) and other antidepressant (*n* = 277, 34%).

**Table 1.** Antidepressants for which tapering strips had been prescribed.

	Frequency	Percentage
Agomelatine	1	0.12
Amitriptyline	16	1.94
Bupropion	25	3.03
Citalopram	87	10.56
Clomipramine	4	0.49
Fluoxetine	28	3.40
Fluvoxamine	8	0.97
Mirtazapine	44	5.34
Nortriptyline	5	0.61
Paroxetine	206	25.00
Sertraline	59	7.16
Venlafaxine	341	41.38
Total	824	100.00

Univariable and multivariable logistic regression, yielding odds ratios (ORs) and 95% confidence intervals (CIs), was conducted to examine associations between dichotomous success of tapering the antidepressant medication as dependent variable and other variables as independent variables. As there were no large or significant interactions with the three categories of antidepressant medication for any variable, results are shown for all antidepressant medications combined. As association with previous withdrawal only applied to the subgroup of individuals with previous attempts at discontinuation (*n* = 577, 71%; Table 3), this association was tested in a separate logistic regression model for this subgroup, including the same variables.

To examine to what degree the sample was representative for adult users of an antidepressant in the Netherlands, we compared the age and sex distribution of the sample with that of antidepressant users in the NEMESIS-2 (Netherlands Mental Health Survey and Incidence Study) sample, a four-wave follow-up of a representative adult general population cohort in the Netherlands (*n* = 6646, aged 18–65 years at baseline), conducted over the period 2007–2018.^22^

## Results

**Figure 1.**
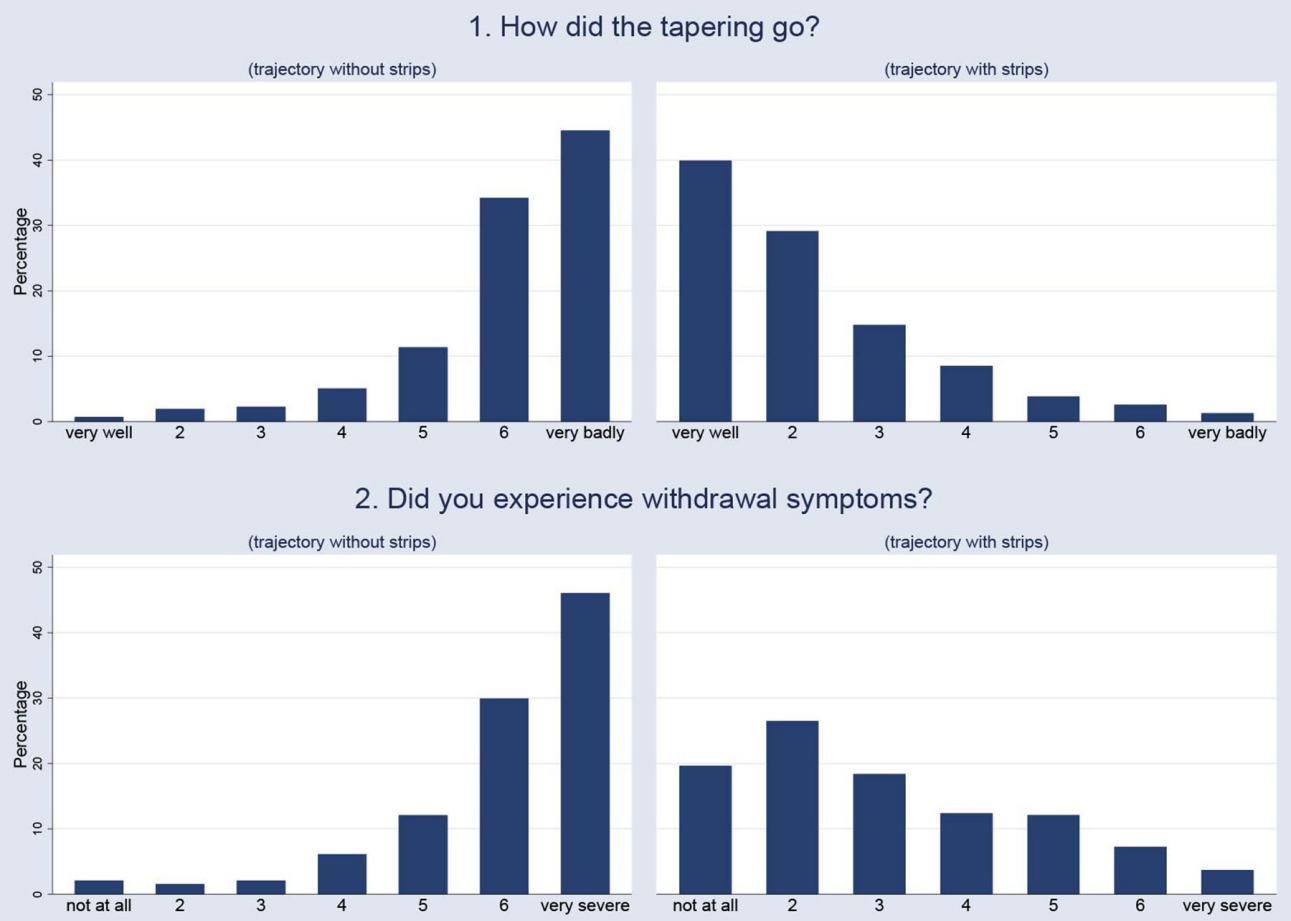
Comparison of tapering trajectories without tapering strips (left) and with tapering strips (right), on outcomes of tapering and severity of withdrawal.

Mean age was 49.5 years, 75% were females, 29% had used tapering strips in their first attempt at discontinuation of antidepressant medication. The sample was demographically representative of adult antidepressant users as mean age of users of an antidepressant in the NEMESIS-2 sample was 49.5 years and 69% were females. Two-thirds of tapering trajectories (66%) were for venlafaxine and paroxetine (Table 1). Most patients had heard of tapering strips from a source other than their prescribing physician; the most frequent single source was Internet (34%; Table 2). The paroxetine group had the largest proportion of long-term users (45% used longer than 10 years; Table 3) and the highest number of previous attempts (25% had had three or more attempts; Table 3). The paroxetine group also used more tapering strips in the trajectory and had a longer tapering trajectory (Table 4). The proportion of successful tapers was lower with more previous attempts (76% first attempters versus 57% in those with more than three attempts; Table 5). A similar trend was seen for duration of antidepressant use (78% successful tapering in those using for less than 1 year, 59% in those using for more than 10 years; Table 5). The proportion of females was higher in the group whose tapering trajectory was not successful (77%) compared to those tapering successfully (70%), as was mean number of tapering strips used, mean number of days of using tapering strips and level of current, but not previous withdrawal (Table 6). Median values were not dissimilar between the groups tapering successfully and not successfully (Table 6). The distribution of values of how well tapering trajectories went without tapering strips (median value = 6, where 7 = ‘very badly’) is the mirror image of the distribution of values of how well the tapering trajectory went with tapering strips (median value = 2, where 1 = ‘very good’; Figure 1). A similar contrast was apparent, albeit somewhat flatter in the tapering strip group, in the comparison between severity of withdrawal experienced without (median severity = 6) and with tapering strips (median severity = 3; Figure 1).

**Table 2.** Tapering strips: source of information.

	*N*	%
Psychiatrist	153	18.87
General practitioner	164	20.22
Internet	276	34.03
Cinderella^a^	32	3.95
Psychologist	21	2.59
Peers	8	0.99
Family/friends	39	4.81
Newspaper, radio, TV	80	9.86
Other	38	4.69
Total	811	100.00

a Non-for-profit pharmaceutical foundation for ‘orphan’ medications.

**Table 3.** Table antidepressant type in relation to duration of use and previous discontinuation attempts.

Antidepressant		Antidepressant duration of use					
		< 1 year	1–2 years	2–5 years	5–10 years	> 10 years	Total
Venlafaxine	*N*	27	49	92	86	87	341
	%	7.92	14.37	26.98	25.22	25.51	
Paroxetine	*N*	5	30	41	38	92	206
	%	2.43	14.56	19.90	18.45	44.66	
Other	*N*	30	54	68	59	66	277
	%	10.83	19.49	24.55	21.30	23.83	
Total	*N*	62	133	201	183	245	824
	%	7.52	16.14	24.39	22.21	29.73	
Antidepressant		Previous discontinuation attempts					
		0	1	2	3	> 3	Total
Venlafaxine	*N*	100	108	85	31	13	337
	%	29.67	32.05	25.22	9.20	3.86	
Paroxetine	*N*	55	58	41	28	22	204
	%	26.96	28.43	20.10	13.73	10.78	
Other	*N*	84	84	58	30	19	275
	%	30.55	30.55	21.09	10.91	6.91	
Total	*N*	239	250	184	89	54	816
	%	29.29	30.64	22.55	10.91	6.62	100.00

**Table 4.** Demographic and tapering variables, stratified by antidepressant type.

Successful tapering^a^		Age	Previous withdrawal^b^	Current withdrawal^c^	Days using tapering strips	Number of strips used	Female sex	First attempters^d^
Venlafaxine	Mean	49.64	6.09	3.08	61.01	2.18	78%	30%
	SD	13.82	1.32	1.68	45	1.61		
	Median	51	7	3	56	2		
	Min	20	1	1	0	0		
	Max	84	7	7	336	12		
	*N*	341	235	304	341	341	341	337
Paroxetine	Mean	51.95	5.90	3.36	70.95	2.53	69%	27%
	SD	13.80	1.39	1.77	39.27	1.40		
	Median	54.5	6	3	56	2		
	Min	21	1	1	28	1		
	Max	80	7	7	252	9		
	*N*	206	147	162	206	206	206	204
Other	Mean	47.54	5.92	2.87	66.28	2.37	75%	31%
	SD	15.09	1.33	1.67	41.71	1.49		
	Median	48	6	2	56	2		
	Min	15	1	1	0	0		
	Max	81	7	7	252	9		
	*N*	277	189	236	275	275	277	275

*N*, total number of individuals; SD, standard deviation.

a Defined as off medication after completion of tapering strips.

b Severity withdrawal experienced in the group with previous attempts before using tapering strips.

c Severity withdrawal experienced during the use of tapering strips.

d Subgroup who never attempted to come off medications before.

**Table 5.** Successful tapering as a function of previous discontinuation attempts and duration of antidepressant use.

Previous discontinuation attempts		Successful tapering		
		Not successful	Successful	Total
0	*N*	56	175	231
	%	24.24	75.76	
1	*N*	57	180	237
	%	24.05	75.95	
2	*N*	56	122	178
	%	31.46	68.54	
3	*N*	27	60	87
	%	31.03	68.97	
> 3	*N*	23	30	53
	%	43.40	56.60	
Total	*N*	219	567	786
	%	27.86	72.14	
Antidepressant duration of use (years)		Successful tapering		
		Not successful	Successful	Total
<1	*N*	13	47	60
	%	21.67	78.33	
1–2	*N*	24	108	132
	%	18.18	81.82	
2–5	*N*	43	147	190
	%	22.63	77.37	
5–10	*N*	48	126	174
	%	27.59	72.41	
> 10	*N*	97	140	237
	%	40.93	59.07	
Total	*N*	225	568	793
	%	28.37	71.63	

**Table 6.** Demographic and tapering variables, stratified by successful tapering.

Successful tapering^a^		Age	Previous withdrawal^b^	Current withdrawal^c^	Days using tapering strips	Number of tapering strips	Female sex	First attempters^d^
No success	Mean	49.73	6	3.54	76.75	2.74	80%	26%
	SD	14.24	1.39	1.92	52.60	1.88		
	Median	51	6.5	3	56	2		
	Min	18	1	1	0	0		
	Max	82	7	7	336	12		
	*N*	225	160	107	224	224	225	219
Success	Mean	49.40	5.97	2.96	60.93	2.18	73%	31%
	SD	14.35	1.34	1.63	36.78	1.31		
	Median	51	6	3	56	2		
	Min	15	1	1	0	0		
	Max	84	7	7	252	9		
	*N*	568	390	566	568	568	568	567

*N*, total number of individuals; SD, standard deviation.

a Defined as off medication after completion of tapering strips.

b Severity withdrawal experienced in the group with previous attempts before using tapering strips.

c Severity of withdrawal experienced during the use of tapering strips.

d Subgroup who never attempted to come off medications before.

Severity of withdrawal while using tapering strips was weakly associated with duration of antidepressant use (Pearson’s *r* = 0.15, *p* < 0.001).

### Risk factors

In the univariable regression analyses, the odds of successful tapering was negatively associated with female sex (OR = 0.672, 95% CI: 0.462–0.978), duration of use (OR linear trend = 0.694, 95% CI: 0.603–0.798), number of previous attempts (OR linear trend = 0.824, 95% CI: 0.725–0.937), severity of withdrawal with tapering strips (OR linear trend = 0.825, 95% CI: 0.733–0.928), use of paroxetine versus reference venlafaxine (OR = 0.496, 95% CI: 0.335–0.737) and use of other antidepressant versus venlafaxine (OR = 0.611, 95% CI: 0.423–0.886). Thus, for each increase in level of length of use of an antidepressant, the probability of successful stopping was reduced multiplicatively by a factor 0.694, that is, a factor 0.232 between lowest and the highest category. Similarly, for each increase in number of previous attempts, the probability of successful stopping was reduced multiplicatively by a factor 0.824, that is, a factor 0.461 between lowest and the highest category.

No associations were apparent for age (OR linear trend = 0.998, 95% CI: 0.988–1.010) and severity of withdrawal without tapering strips (OR linear trend = 0.982, 95% CI: 0.856–1.127).

Successful tapering was associated with using less tapering strips (OR linear trend 0.791, 95% CI: 0.716–0.875) and a shorter tapering trajectory in days (OR linear trend = 0.992, 95% CI: 0.988–0.995).

In the multivariable regression, these associations were retained, with the exception of the association with number of previous attempts and paroxetine (Table 7). In the subgroup analysis of those with a previous attempt, no association was apparent for severity of previous withdrawal (OR = 1.0620, 95% CI: 0.883–1.279), consistent with the univariable analysis.

**Table 7.** Multivariable logistic regression table (*N* in model = 672).

Success of tapering trajectory^a^	OR	*p*	95% Confidence interval	
Age in years	0.998	0.838	0.982	1.015
Sex				
Male^b^	1.00			
Female	0.502	0.017	0.284	0.886
Duration of use of antidepressant medication (years)				
< 2^b^	1.00			
2–5	0.725	0.418	0.333	1.579
5–10	0.444	0.033	0.210	0.937
> 10	0.243	0.000	0.118	0.498
OR linear trend^c^	0.618	0.000	0.494	0.774
Number of previous attempts				
0^b^	1.00			
1	1.624	0.131	0.866	3.045
2	0.787	0.434	0.433	1.432
3	1.264	0.552	0.585	2.733
> 3	0.807	0.614	0.352	1.854
OR linear trend^c^	0.933	0.455	0.779	1.118
Level of withdrawal experienced with tapering strip^d^	0.851	0.011	0.751	0.963
Type of antidepressant				
Venlafaxine^b^	1.00			
Paroxetine	0.736	0.297	0.414	1.309
Other	0.508	0.009	0.305	0.846

OR, odds ratio.

a Defined as off medication after completion of tapering strips.

b Reference category.

c The summary increase in odds with one unit change in the exposure variable (duration of use and previous attempts).

d Odds ratio linear trend for severity withdrawal experienced during the use of tapering strips (1–7 Likert scale).

## Discussion

### Findings

Results are summarised in Box 1. The current analysis represents the third replication of the real-world effectiveness – a 72% success rate – of tapering strips for discontinuation of antidepressant medication. The results of this study are remarkably similar to those of two previous studies.^20,21^ Duration of antidepressant use, number of previous attempts, antidepressants other than venlafaxine, female sex and more severe withdrawal during the tapering trajectory reduced the odds of tapering success. Multivariable regression suggested the association with paroxetine and the association with number of previous attempts was reducible to other factors in the model.

**Box 1.** Short summary of results of this study and level of replication across two previous studies^20,21^ (see also Table 8).

Background: Physicians often struggle to help patients taper antidepressants safely because the current practice of tapering is not informed by evidence and lower dosages required for safe tapering are not readily available. To address these problems, tapering strips were developed to implement the Horowitz–Taylor personalised hyperbolic method of tapering to prevent withdrawal. Efficacy was investigated in this and two earlier studies with a total of 2127 participants who were demographically representative of antidepressant users in the Netherlands but likely were selected for having withdrawal at the severest end of the spectrum. ● Most participants were long-time users, many of whom had tried to taper previously: 8% had used antidepressants less than 1 year (40% 1–5 years, 22% 5–10 years and 30% more than 10 years); 70% had tried to taper previously (consistent across studies). ● Tapering strips were prescribed majorly (60–80%) for venlafaxine and paroxetine (consistent across studies). ● Discontinuation of SSRIs with longer half-lives may also cause severe withdrawal requiring use of tapering strips, albeit probably less frequently. ● 70% tapered completely using tapering strips; whether or not they had tried to taper in the past made no difference (replicated across studies). ● Patients rated tapering with tapering strips as much easier (standardised effect size > 2; replicated across studies). ● Patients rated tapering with tapering strips as occurring with much less withdrawal (standardised effect size > 1.5; replicated across studies). ● Of participants who had tapered successfully using tapering strips, 72% was still without antidepressant 1–5 years later (follow-up data study 2)^21^. ● The best replicated risk factor for failure to taper medication was longer use of antidepressants; weaker risk factors were more previous attempts to discontinue medication, less withdrawal during previous attempts, more withdrawal using tapering strips and female sex. ● Paroxetine may be more difficult drug to taper than other antidepressant medications.

Given the fact that patients requiring prescription for a tapering strip can be considered as the group most severely affected – confirmed by the high rate of previous attempts, long period of use of antidepressant medication and the fact that their doctor had decided to prescribe a tapering strip in the first place, the results can be interpreted as evidence that tapering strips are effective for those at the highest end of the severity spectrum of antidepressant withdrawal.

Paroxetine was the third, and venlafaxine the fifth most commonly prescribed antidepressant in the Netherlands in 2019 (source: vektis.nl). The fact that they were by far the most common antidepressants that tapering strips were requested for in this and previous surveys^20,21^ suggests, given over-representation in the study group relative to overall prescription rates, that use of these medications is associated with greater risk of withdrawal.

The external validity of research results for a public health problem, such as medication withdrawal is important. About 70% of patients in this study were able to taper completely when they used tapering strips. This was the case for patients who tapered for the first time as well as for the 71% who had tried to taper previously without tapering strips. Although the group was restricted to those with the highest levels of previous withdrawal, they were demographically representative of adult users of antidepressants in the Netherlands (albeit with likely overrepresentation of paroxetine and venlafaxine users) and all tapers occurred in routine clinical practice without the typical exclusion of many patients as routinely occurring in RCTs (randomized controlled trials).^23^

### Comparison with previous tapering studies

The results of the three questionnaire-based retrospective cohort studies with tapering strips to date are summarised in Table 8. As the results are highly consistent across three independent studies, it must be considered strong evidence for the effectiveness of tapering strips in the real world.^24^ The risk factor with particularly strong evidence is duration of use of antidepressant medication. This is an important observation, given evidence that long-term use of antidepressants is a recognised risk factor for withdrawal.^25,26^ The mechanisms for the relationship between longer use and more difficulties tapering antidepressants are not known. Even short-term use of antidepressants induces a range of brain and metabolic changes^27^ and there is meta-analytic evidence suggesting that chronic use of medication may make patients more relapse-prone.^28^ It has been pointed out that continued drug treatment with antidepressant medications may stimulate processes that represent a reversal of the initial acute withdrawal effects of a drug.^7^ The ‘oppositional model of tolerance’ has been invoked to explain loss of treatment efficacy during maintenance treatment and the fact that some side effects tend to occur only after a certain time. These processes may also steer the mental disorder into a development of treatment-unresponsiveness, which may also include expressions of bipolar disorder or paradoxical reactions. When drug treatment ends, oppositional processes are no longer kept in check by resistance, resulting in a range of possible outcomes including new withdrawal symptoms, possible persistent post-withdrawal disorders (the existence of which has not been established firmly, more research being required), hypomania and resistance to treatment if it is reinitiated.^7^

**Table 8.** Results of current and two previous tapering strip studies^20,21^ combined.

Exposures and outcomes	Study 1^20^	Study 2^21^	Study 3^a^
Survey response rate (%)	68	48	59
Number of patients wishing to discontinue antidepressants (*N*)	895	408	824
Female sex (%)	69	70	75
Age (mean)	50	52	50
Proportion venlafaxine (%)	43	47	41
Proportion paroxetine (%)	47	35	25
Received information on tapering strips from non-medical source (%)	60	–	61
Had previously tried unsuccessfully (%)	62	61	71
Severity withdrawal without tapering strip^b^ (mean)	6.1	–	6.0
Severity withdrawal with tapering strip^b^ (mean)	3.2	–	3.1
Number of withdrawal symptoms without tapering strip (from 0 to 8; mean)	–	3.4	–
Number of withdrawal symptoms with tapering strip (from 0 to 8; mean)	–	1.1	–
Length of follow-up after tapering trajectory (years)	^c^	1–5	^c^
Number of tapering strips used (median)	2	–	2
Number of days of use of tapering strips (median)	56	–	56
Success rate^d^ (%)	71	66	72
Success rate^d^ subgroup ‘first attempt’ (%)	73	68	76
Success rate^d^ subgroup ‘attempted previously’ (%)	70	65	71
Longer use of antidepressant	+	+	+
More previous attempts	+	–	+
Less withdrawal previous attempts	+	+	–
More withdrawal with use of tapering strips	–	–	+
Paroxetine versus other	±	–	+
Female sex	–	–	+

a This study.

b Rated on a 1–7 Likert scale.

c No follow-up after tapering, only period of tapering itself and the outcome of tapering.

d Defined as off medication after completion of tapering strips.

### The importance of personalised tapering

Although 72% remained off medication after using tapering strips, around one-third of the sample did not meet the pre-set definition of ‘successful tapering’. While some of these individuals likely will be able to stop their medication later, they were not successful in the short term. As described elsewhere, rate of tapering can be a very important factor and some individuals may be able to come off medications if a slower and more personalised approach is adopted.^9^ In addition, shared decision-making is crucial. Optimising these may help persons come off antidepressants even if previous tapering was not successful. Self-monitoring can sometimes add an extra dimension, making the tapering more personalised and more robust to manage early withdrawal.^9,29^ The most effective way to tackle the problem, however, is prevention, using psychotropic medications more conservatively and taking into account the fact that some compounds likely are more associated with withdrawal than others. A powerful rational for prevention comes from surveys of antidepressant users indicating that 30–50% of long-term antidepressant prescriptions had no evidence-based indication.^30^

It is important to note that not being able to taper completely in the short term does not necessarily imply ‘failure’ for the patient. Our study could not inform on how many patients were able to reduce their daily dose to their satisfaction, how many could have tapered completely, had they taken more time to taper more gradually or if they had been able to stabilise during tapering when withdrawal symptoms appeared. Indeed some may have been satisfied despite the fact that tapering failed, because they had more control over the tapering process. For example, 50% of patients who did not meet the criterion for successful tapering still rated the question ‘how well did discontinuation with tapering strips go’ as good or very good.

A total of 28 individuals in the sample indicated they had used tapering strips for fluoxetine, which theoretically is assumed to less predictive of withdrawal given its long half-life. Of the 19 individuals who had indicated they had previously tried to come off fluoxetine, however, median severity of withdrawal had been 6, the same as in the paroxetine and venlafaxine groups. In other words, the half-life of a compound likely may not be the only or even the main factor driving likelihood of withdrawal.

Using less tapering strips and a shorter tapering trajectory were associated with more success. While this may represent a chance finding, one possibility is that the group of people who choose for a shorter tapering trajectory may ‘know’, or rightly suspect, that this will be sufficient to taper safely. Moreover, the group that expects more problems – perhaps on the basis of previous or current bad experiences – rightly chooses for longer trajectories. If true, this would certainly present an argument for careful shared decision-making in deciding on the length of tapering.

### Future research

To date, only a limited number of studies have focused on approaches to discontinuation of antidepressants after long-term use. Unfortunately, most, if not all of these studies were not free of withdrawal confounding,^30^ making it difficult to draw conclusions from these. In addition, the combined very low-certainty results of these studies must be considered unimpressive at best and add little to improving routine clinical practice.^30^

It is often assumed that the best way to examine the efficacy of tapering strips is to compare them to ‘regular tapering’ in a randomised controlled trial. However, such a comparison is problematic as currently no definition exists of what would constitute ‘regular tapering’ and it is not known how to accommodate extensive patient heterogeneity in how medication reduction is best titrated against severity of withdrawal. Indeed, the whole concept of ‘regular tapering’ does not appear to exist as the current practice is that patients and doctors are improvising in a variety of patient- and doctor-specific ways to facilitate the tapering of medication in patients experiencing significant withdrawal.^3^ It may also prove difficult, for example, in the case of venlafaxine for which small doses do not exist, to randomise patients to a ‘regular tapering’ arm, given widespread access to venlafaxine tapering strips for a personal and flexible tapering trajectory.

### Methodological issues

The results should be interpreted in the light of the following methodological issues.

The number of tapering strips patients used to taper is not indicative for/cannot inform about the total length of a tapering trajectory because the data cannot provide information on if and how much patients had already tapered from a given starting dose before they started using tapering strips. The fact that over the period of observation, most Dutch health insurers would not reimburse tapering medication will have influenced decisions of doctors and patients about how to taper.

The sample was largely selected by the referring physician for unsuccessful previous tapering and chronic use of antidepressant medications. Although 29% were first-attempters, these patients may represent a subgroup with risk factors in addition to chronic use, for example, experiencing withdrawal symptoms when forgetting to take the medication for a few days (information that was not available in the survey). In other words, the reported 72% rate of successful tapering using tapering strips applies to the selected group considered most at risk of severe withdrawal and difficulties coming off antidepressant. The external validity of the research, therefore, may extend to the group of people most at risk of withdrawal in countries with comparable rates of antidepressant prescription.

Around 60% of the sample responded, with the possibility of bias due to selective non-response. However, even if the non-responder success rate had only been half that of the observed success rate, the overall rate in the entire sample would still be around 57%, which may still be considered a high percentage for the group most at risk of severe withdrawal and previous unsuccessful attempts at discontinuation. Furthermore, as this was the third independent replication in as many independent samples, it is highly unlikely that a similar non-differential attrition would occur three times in a row.

To increase the response rate, the quality assurance questionnaire was kept brief, preventing us from more precise assessment of variables, for example, nature and type of relapse, relationship with and support by prescriber, total length of tapering trajectories (including the part carried out without the use of tapering strips), current level of symptoms, and so on.

Assessment of withdrawal, similar to pain and low mood, is subjective. However, the main interest of the current analysis was the within-patient comparison of withdrawal while tapering with or without tapering strips, that is, within the same patient-specific anchors of withdrawal experience. Recall of previous withdrawal when attempting to discontinue an antidepressant without the use of tapering strips may be influenced by the relative success of less withdrawal with the more recent attempt using a tapering strip, possibly inflating effect sizes of the comparison between previous and current withdrawal. This issue, therefore, needs to be replicated in prospective work.

**Table:** Glossary of terms used in this publication

Tapering	Gradual dose reduction
Linear tapering	Constant dose reduction by a similar amount at each step, resulting in hyperbolic reduction of receptor occupancy
Hyperbolic tapering	Dose reduction in unequal steps which become smaller and smaller as the dose gets lower and lower
Tapering schedule	Dose reduction schedule from a given dose to a lower dose (which can be zero) over a certain period of time
Personalised tapering	Tapering using an individualised tapering schedule, to be flexibly adapted when required, conducted with tapering medication or another form of dose reduction
Tapering strip	Strip containing medication for 28 days, packaged in a roll or strip of small daily pouches. Each pouch is numbered and has the same or slightly lower dose than the one before it. Dose and day information on each pouch allow patients to precisely record and monitor the progress of their dose reduction
Tapering trajectory	Part of a taper completed using one or more tapering strips^a^
Horowitz–Taylor method for tapering of psychiatric medication	Method for personalised hyperbolic tapering to achieve gradual linear reduction of receptor occupancy in order to prevent withdrawal
Receptor occupancy	Occupancy of the receptor on which a drug acts (for antidepressants the serotonin transporter, for antipsychotics the dopamine receptor and so on)
Stabilisation	Letting a patient stay on a given dose, instead of continuing the reduction schedule, when withdrawal occurs during tapering or for other reasons (e.g. patient anxiety)
Stabilisation strip	(tapering) strip in which the dose – which can be any dose – remains the same
Withdrawal	Withdrawal symptoms^a^; discontinuation of a drug
Current withdrawal^a^	Withdrawal when using a tapering strip
Previous withdrawal^a^	Withdrawal during previous discontinuation attempts without using a tapering strip

a This study.
